# In the quest for degenerative lumbar spinal stenosis etiology: the Schmorl’s nodes model

**DOI:** 10.1186/s12891-017-1512-6

**Published:** 2017-04-20

**Authors:** Janan Abbas, Viviane Slon, Dan Stein, Natan Peled, Israel Hershkovitz, Kamal Hamoud

**Affiliations:** 1 Department of Anatomy and Anthropology, Sackler Faculty of Medicine, Tel Aviv University, Tel Aviv, Israel; 2 Department of Physical Therapy, Zefat Academic College, Zefat, Israel; 3 Department of Radiology, Carmel Medical Center, Haifa, Israel; 4 Faculty of Medicine in the Galilee, Bar-Ilan University, Zefat, Israel; 5 Department of Orthopaedic Surgery, The Baruch Padeh Poriya Medical Center, Tiberias, Israel

**Keywords:** Degenerative lumbar spinal stenosis, Schmorl’s nodes, Computerized tomography (CT)

## Abstract

**Background:**

Degenerative lumbar spinal stenosis (DLSS) is a common health problem in the elderly and usually associated with three-joint complex degeneration. Schmorl’s nodes (SNs) are described as vertical herniation of the disc into the vertebral body through a weakened part of the end plate that can lead to disc degeneration. Since SNs can harm the spine unit stability, the association between DLSS and SNs is expected. The aim of this study is to shed light on the relationship between degenerative lumbar spinal stenosis and SNs.

**Methods:**

Two groups of individuals were studied: the first included 165 individuals with DLSS (age range: 40–88, sex ratio: 80 M/85 F) and the second 180 individuals without spinal stenosis related symptoms (age range: 40–99, sex ratio: 90 M/90 F). The presence or absence of SNs on the cranial and caudal end plate surfaces at the lumbosacral region (from L1 to S1 vertebra) was recorded, using CT images (Brilliance 64 Philips Medical System, Cleveland Ohio, thickness of the sections was 1–3 mm and MAS, 80–250). Chi-Square test was taken to compare the prevalence of SNs between the study groups (control and stenosis) by lumbar disc level, for each gender separately. Multivariable logistic regression analysis was also used to determine the association between DLSS and SNs.

**Results:**

The prevalence rate of SNs was significantly greater in the stenosis males (L1-2 to L5-S1) and females (L4-5 and L4-S1) compared to their counterparts in the control (*P <* 0.001). In addition, the presence of SNs in both males and females was found to increase the likelihood for DLSS.

**Conclusions:**

Our results indicate that SNs prevalence is significantly greater in the DLSS group compared to the control. Furthermore, SNs are strongly associated with DLSS.

## Background

Degenerative lumbar spinal stenosis (DLSS) is a common health problem in the elderly population and the most frequent indication for spinal surgery in individuals over 60 years [1, 2]. The clinical prevalence of this condition is approximately 47% in adults with symptoms of pain and numbness referred to the lower extremities [3].

DLSS is essentially associated with degenerative changes of the three-joint complex (intervertebral disc anteriorly and 2-facet joints posteriorly), ligamentum flavum (LF) thickening and osteophyte formation [4–7]. It is well accepted that DLSS usually begins at the intervertebral disc (e.g. disc height loss) that may cause an instability of the spine segment [8, 9] leading in time to degenerative cascade of the spine unit.

Schmorl’s nodes (SNs) have been described as herniation of the intervertebral disc into the vertebral body through an area of weakness in the endplate [10, 11]. The etiology of SNs is still unknown, although an association with trauma to spine, infection, genetics and several diseases (basically metabolic) was found [10, 12, 13]. SNs are common in human spine mainly in the lower thoracic and lumbar regions [14] which are attributed to the high load applied on these vertebrae [10, 15]. The prevalence of SNs has a great range with those evaluated from cadaveric spine being much higher than those acquired from radiological images [12, 14, 16, 17]. Although CT scan might be a good modality for observing and detecting SNs [18], there have been very few investigations using methodology based on this modality, since this could expose the participants to redundant radiation.

SNs are usually asymptomatic [11, 14]; however, some studies have suggested a direct relationship between the presence of these nodes and back pain [19, 20]. Previous studies have reported a positive correlation between SNs and lumbar disc disease [19]. Mok et al. [21] have also shown that SNs were correlated with increased severity of disc degeneration.

Because SNs can cause lumbar segment instability [21] (e.g., disc height loss), we hypothesized that (a) SNs are more common in DLSS population and (b) the location of these nodes will differ in males and females due to variation in their lumbar spine postures [22]. The first hypothesis is further supported by the findings that both vertical and horizontal herniations add strain to the posterior ligaments, ligamentum flavum included, as well as to the zygapophyseal joints, ensuing increased pressure on the spinal canal [17].

The aims of the current study are to reveal the prevalence and locations of SNs in the DLSS population, in order to shed light on the pathophysiology of this phenomenon.

## Methods

### Study design and groups

**Fig. 1:**
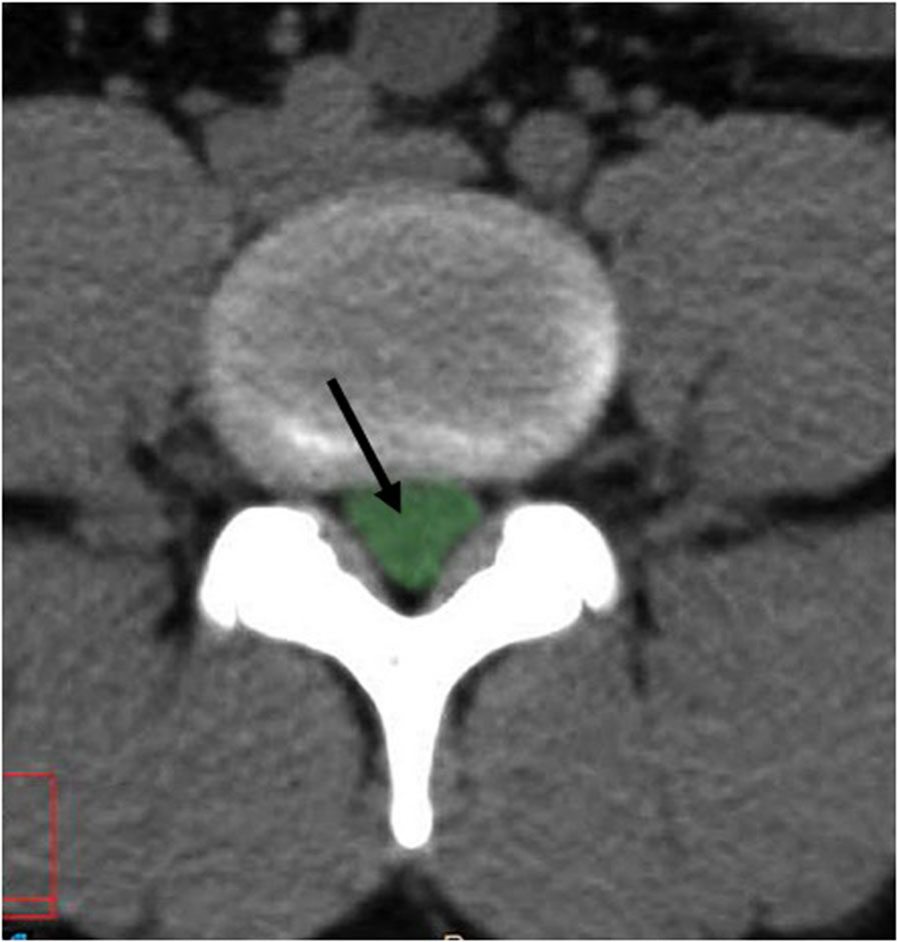
Measurement of lumbar cross-sectional area of the dural sac

This study was conducted as a cross-sectional retrospective study with two groups of individuals. The first group was a control group that included 180 individuals without spinal stenosis related symptoms (age range: 40–99 years, sex ratio: 90 M/90 F). All were randomly collected from a pool of subjects referred to the Department of Radiology, from 2008 to 2010 for abdominal CT scans due to renal colic symptoms. The second was the DLSS group which included 165 individuals (age range: 40–88 years; sex ratio: 80 M/85 F), who were enrolled from 2006 to 2010 and interviewed by a spine surgeon (K.H). All had intermittent claudication accompanied by other symptoms related to spinal stenosis (LBP and radicular referred pain) [23–25]. CT scans of the DLSS group were interpreted by the same surgeon (K.H) and all exhibited a reduced cross-sectional area (CSA) of the dural sac (<100 mm^2^) [25–28] of at least one lumbar level. As described previously [29], the CSA was measured in the axial plane at the lumbar intervertebral disc level (Fig. 1), using CT images (Brilliance 64 Philips Medical System, Cleveland Ohio, thickness of the sections was 1–3 mm and MAS, 80–250). This workstation enabled the processing of the scans in all planes and allowed a 3D reconstruction of the lower lumbar region. Individuals under 40 years of age as well as those with congenital stenosis (AP diameter of the bony canal < 12 mm) [23, 30], fractures, spondylolysis, tumors, Paget’s disease, steroid treatment, severe lumbar scoliosis (>20°) and iatrogenic (post laminectomy, post fusion) were excluded from this group. All CT images for both groups were taken in the supine position with extended knees.

**Fig. 2:**
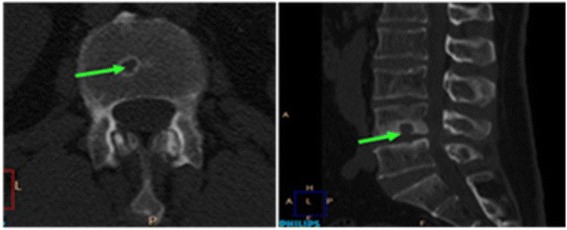
Schmorl’s node as seen on CT scan. Axial (left) and sagittal (right) sections

The presence or absence of Schmorl’s nodes on the cranial and caudal endplate surfaces at the lumbosacral region (from L1 to S1 vertebra) was recorded. Both axial and sagittal planes were utilized for this purpose (Fig. 2). Schmorl’s node was defined as a focal lesion in the vertebral endplate usually with sclerotic margins [31]. Lesions that were deeper than 2 mm were considered in the current study.

The study populations were divided into 2 sub-groups, with (SN group) and without SNs (non-SN group). Location of SNs was determined based on disc level (e.g. L2-L3) rather than specific discal surfaces (e.g., superior discal surface of L3), this in order to reveal any possible relationship between SNs and spinal segment disorder (e.g. canal stenosis).

As described previously [29], all participants, both in the DLSS and the control groups, were interviewed either in the spine clinic or radiology department, as a part of their intake procedure. The interviews were used to obtain data on body size (e.g. height, weight and BMI) and systemic diseases (hypertension- HTN and/or diabetes mellitus- DM) from the patients as well as on demographical aspects such as occupation (e.g., engaged in heavy manual labor), smoking habits and number of deliveries. Individuals were recognized as having HTN and/or DM if their oral reports during the interview coincided with their medical reports (receiving anti-hypertensive and/or diabetes mellitus medications). BMI was calculated as the ratio of body mass (in kg) divided by height in meters squared. Occupation was divided into four categories: (a) heavy manual labor, (b) housekeeping, (c) work requiring prolonged sitting and (d) other. All participants were also classified as smokers or non-smokers. Individuals who smoked ≥ 10 cigarettes per day for at least five years were classified as smokers.

#### Statistical analysis

Chi-Square test was taken to compare the prevalence of SNs between the study groups (control and stenosis) by lumbar disc level, for each gender separately. To identify the relationships between SNs and degenerative lumbar spinal stenosis, we used a multivariable logistic regression analysis after adjusting for all demographics, life-style and health data variables that could act as confounding factors and affect the interaction between SN and DLSS (dependent variable- DLSS; independent variable; SN, age, height, weight, BMI, number of deliveries, heavy manual labor, smokers, hypertension and/or diabetes mellitus).

Kappa coefficient was calculated to determine the intra-tester and inter-tester reliability of SNs (repeated measurements of 20 individuals). Intra-tester reliability was assessed by one of the authors (JA) who examined the SNs presence twice within intervals of 3–5 days. Inter-tester reliability involved two testers (JA and KH), who took the measurements within an hour of each other. Both testers were blinded to the results of the measurements. Significant difference was set at *P <* 0.05.

## Results

Both intra- and inter-tester reliability rates were very high; 0.94 and 0.90 respectively.

No significant difference was found in the mean age between the control males and females and their counterparts in the stenosis group (Table 1).

**Table 1:** Mean age ± standard deviation (SD) of the control and the stenosis groups by gender. *N =* sample size

Study groups	Mean age (years) ± SD	*P* Value
Control males (*N =* 90)	62.9 ± 12.38	0.066
Stenosis males (*N =* 80)	66.2 ± 10.82	
Control females (*N =* 90)	62 ± 12.97	0.795
Stenosis females (*N =* 85)	62.5 ± 8.63	

### Prevalence of Schmorl’s nodes in the study groups

From a total sample of 345 individuals, (control and DLSS) 202 individuals (58.5%) manifested at least one or more SNs along the lumbosacral vertebrae (L1 to S1). In the DLSS group (*n =* 165), 122 exhibited SNs (73.9%), whereas in the control group (*n =* 180), 80 individuals had SNs (44.4%) (*P <* 0.001).

**Fig. 3:**
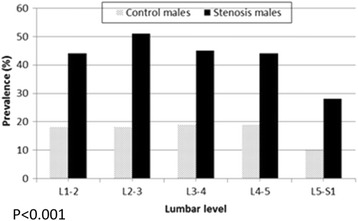
Prevalence (%) of Schmorl’s nodes in both study groups (control and stenosis) by lumbar levels, males only

**Fig. 4:**
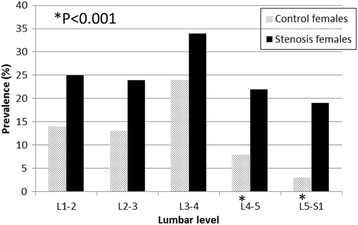
Prevalence (%) of Schmorl’s nodes in both study groups (control and stenosis) by lumbar levels, females only

The prevalence rate of SN by lumbar disc levels in both study groups for males and females is illustrated in Figs. 3 and 4.

The prevalence of SN was significantly greater in the stenosis males (L1-2 to L5-S1) and females (L4-5 and L4-S1) compared to their counterparts in the control (*P <* 0.001) (Figs. 3 and 4).

### Association between Schmorl’s nodes and degenerative spinal stenosis

After adjusting for possible confounding factors (e.g. age, BMI, heavy manual labor), it was found that SNs in males, at L2-3 and L4-5 levels, are significantly associated with DLSS (Table 2). In females, the presence of SN at L4-5 and L5-S1 levels increases the likelihood that this individual will manifest degenerative spinal stenosis.

**Table 2:** Multivariable logistic regression analyses (following adjustment for demographic, lifestyle and health parameters) demonstrating the variables that increase the likelihood of degenerative lumbar stenosis development, males and females separately

	OR	(CI) 95%	*P* value
Males			
SN L2-3	4.665	2.079–10.472	<0.001
SN L4-5	2.624	1.154–5.968	0.021
BMI	1.112	1.022–1.211	0.014
Heavy manual labor	5.738	2.736–12.037	<0.001
Female			
SN L4-5	3.292	1.162–9.330	0.023
SN L5-S1	8.614	2.150–34.510	0.002
No. of deliveries	1.2164	1.102–1.448	0.001

*SN* Schmorl’s node, *OR* Odds ratios, *CI* confidence intervals, *BMI* body mass index

## Discussion

Our findings indicate that the prevalence of SNs is significantly greater in the DLSS group compared to their counterparts in the control group. SNs, BMI and heavy manual labor increase the likelihood for DLSS in the male population and number of deliveries in the female population.

**Fig. 5:**
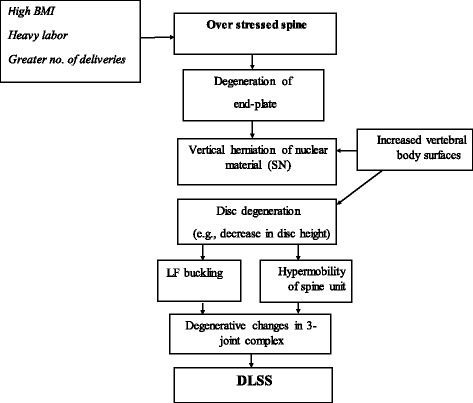
A model that explains the possible association between Schmorls nodes (SNs) and degenerative lumbar spinal stenosis (DLSS)

To our knowledge, the association between SNs and DLSS has not been addressed in previous studies, hence the lack of comparative analysis. Although DLSS is a multifactorial phenomenon, including genetic and environmental factors, we here propose a rational pathway to explain the relationship between DLSS and SNs (Fig. 5).

The association between BMI, manual heavy labor, number of deliveries and DLSS has been established recently [29]. It has been shown that manual work that involves repetitive movements, such as back twisting or bending forwards, poses extreme pressure on the lower spine [32], mainly on the intervertebral disc [33, 34] and facet joints, and therefore can accelerate degenerative spine disease. The continuous loading with higher body weight over the spine could increase the susceptibility of endplates to failure and increase the risk of sustaining SN [35]. It has been found that overweight individuals were more likely to manifest SNs [21] and heavy occupation was associated with the presence of SNs [19]. All these studies are in accordance with the original theory pertaining to Schmorl’s nodes pathophysiology, i.e., that the protrusion of the nucleus through the endplate is mainly due to axial loading [36].

Although we are not aware of any study that has investigated the association between SNs and DLSS, our previous study [37], has suggested that the existence of primary disorder of the vertebral endplate could explain DLSS pathogenesis. Indeed, we did not find any evidence in the English literature to support this notion except for two studies that stated a possible relationships between endplate disorder and abnormal dimensions of the vertebral body and spinal canal [38, 39].

It has been suggested that vertebral body shape may induce the development of intervertebral disc herniation. Pfirrmann and Resnick [14] found in a cadaveric study that Schmorl’s nodes were associated with a straight vertebral endplates compared to more concave ones. In addition, Harrington et al [40] have reported that the size and shape of the vertebral body related with lumbar intervertebral disc herniation. A recent skeletal study [41] showed a correlation between the morphology and size of vertebrae and the presence of Schmorl’s nodes. It is noteworthy that individuals with DLSS also manifested larger vertebral body dimensions [37]. Harrington et al [40] suggested that the diameters of the vertebral disc influence its ability to withstand tension during compression. Their argument rests on LaPlace’s law [42] which states that the ability of a fluid-filled tube wall to withstand tension decreases with increasing radius. We can assume that rounded (cylindrical) vertebral bodies possess larger diameters than the more “kidney-shaped” vertebral bodies, making the vertebral disc more vulnerable to stress, hence the higher rate of disc herniation among individuals with large vertebral bodies. In addition, larger intervertebral discs are correlated with degeneration [43].

SNs are also associated with disc degeneration (DD) and osteophytes formation [13, 14, 19]. It has been reported that protrusion of nucleus pulposus into the vertebral body causes direct loss of nucleus contents leading to DD [44]. A recent study has shown that SNs were associated with severe degeneration and disc height narrowing [21]. This later phenomenon will necessarily lead to buckling of the LF, henceforth, to spinal stenosis. Additionally, disc height loss might render the spine stability (due to extra mobility), imposing greater strain on the posterior elements of the vertebra, inciting the 3-joint complex cascade degeneration (such as facet joint arthrosis) that may eventually lead to DLSS.

In summary, it seems that increased loading on the lumbar spine due to different activities (e.g., lifting heavy objects, repetitive flexion/twisting movements, weight gain during pregnancy and higher BMI in general), may lead to a failure of the endplate. This will be followed by disc herniation (SNs), disc height narrowing, LF buckling and eventually to DLSS. Individuals with larger discs are more prone to develop SNs and or DD, henceforth, DLSS. As spine stability is deteriorating (hypermobility) in the presence of degenerating discs (SNs being a hale mark), degenerative process in other components of the segment motion are accelerated, leading eventually to spinal canal stenosis.

Finally, one of the interesting findings of the current study is the fact that in males SNs at L2-3 and L4-5 levels relate to DLSS, whereas in females these are the SNs at L4-5 and L5-S1 levels. This discrepancy between males and females can partially be explained by their differences in lordosis shape. Hay et al [22] have recently reported that the location of the lumbar curve (lordosis) peak is significantly lower in females compared to males, implying a greater stress on the lower segment motions that may lead in time to degenerative changes and stenosis.

## Clinical implication

Although SNs are usually asymptomatic, physicians should be aware of this phenomenon, when present SNs can increase the likelihood for developing DLSS. We believe that specific exercise intervention based on motor learning model, which was evident for lumbar segmental instability [45], could be useful for individuals with SNs, to prevent or at least to delay the onset of degenerative lumbar stenosis.

### Study limitations

As in any cross-sectional study, no causal relationship between SNs (exposure factors) and DLSS can be determined [46]. In addition, data on demographic and lifestyle variables could include some degree of recall bias. This study has been conducted in a small modern country with relatively small population; that could limit the generalization of the results.

## Conclusions

SNs are strongly associated with DLSS. Over-loading on the lumbar spine (e.g., lifting heavy objects, weight gain during pregnancy and higher BMI) leads to failure of the endplate, followed by disc herniation (SNs). This, in turn, causes reduction in disc height, leading to LF buckling and eventually, to DLSS.

## Abbreviations

AP: Anterior posterior
BMI: Body mass index
CI: Confidence interval
CSA: Cross-sectional area
CT: Computed tomography
DD: Disc degeneration
DLSS: Degenerative lumbar spinal stenosis
DM: Diabetes mellitus
ICCs: Intra-class correlations
LBP: Low back pain
LF: Ligamentum flavum
OR: Odds ratio
SD: Standard deviation
SNs: Schmorl’s nodes
THN: Hypertension
